# Capacitive and Efficient Near-Infrared Stimulation of Neurons via an Ultrathin AgBiS_2_ Nanocrystal Layer

**DOI:** 10.1021/acsami.4c01964

**Published:** 2024-05-29

**Authors:** Ridvan Balamur, Jae Taek Oh, Onuralp Karatum, Yongjie Wang, Asim Onal, Humeyra Nur Kaleli, Cigdem Pehlivan, Afsun Şahin, Murat Hasanreisoglu, Gerasimos Konstantatos, Sedat Nizamoglu

**Affiliations:** † Department of Electrical and Electronics Engineering, Koç University, Istanbul 34450, Turkey; ‡ ICFO-Institut de Ciencies Fotoniques, The Barcelona Institute of Science and Technology, Castelldefels, Barcelona 08860, Spain; § Department of Biomedical Science and Engineering, Koç University, Istanbul 34450, Turkey; ∥ Research Center for Translational Medicine, Koç University, Istanbul 34450, Turkey; ⊥ ICREA - Institució Catalana de Recerca i Estudiats Avançats, Lluis Companys 23, Barcelona 08010, Spain

**Keywords:** nanocrystal, quantum dot, solar cell, photovoltaic, neuron, stimulation, AgBiS_2_, bioelectronic

## Abstract

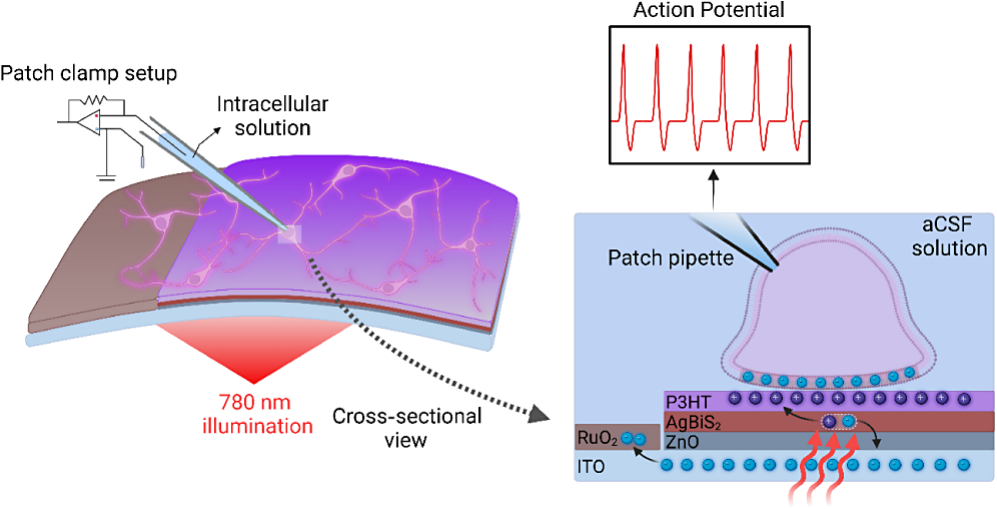

Colloidal nanocrystals (NCs) exhibit significant potential for photovoltaic bioelectronic interfaces because of their solution processability, tunable energy levels, and inorganic nature, lending them chemical stability. Silver bismuth sulfide (AgBiS_2_) NCs, free from toxic heavy-metal elements (e.g., Cd, Hg, and Pb), particularly offer an exceptional absorption coefficient exceeding 10^5^ cm^–1^ in the near-infrared (NIR), surpassing many of their inorganic counterparts. Here, we integrated an ultrathin (24 nm) AgBiS_2_ NC layer into a water-stable photovoltaic bioelectronic device architecture that showed a high capacitive photocurrent of 2.3 mA·cm^–2^ in artificial cerebrospinal fluid (aCSF) and ionic charges over 10 μC·cm^–2^ at a low NIR intensity of 0.5 mW·mm^–2^. The device without encapsulation showed a halftime of 12.5 years under passive accelerated aging test and did not show any toxicity on neurons. Furthermore, patch-clamp electrophysiology on primary hippocampal neurons under whole-cell configuration revealed that the device elicited neuron firing at intensity levels more than an order of magnitude below the established ocular safety limits. These findings point to the potential of AgBiS_2_ NCs for photovoltaic retinal prostheses.

## Introduction

Electrical neurostimulation plays a crucial role in unraveling the mysteries of the nervous system and offering drugless treatments for neurological diseases.^1^ These devices operate by extracellular modulation of the membrane potential through electrical pulses, which either activate or silence neural activity. In this process, an implantable pulse generator sends electronic signals via lead wires to the electrodes, inducing ionic currents that traverse through nerve tissue back to the return electrode.^2^ Over the years, millions of such bioelectronic devices have been successfully implanted in patients, enabling the treatment of diverse conditions including heart failure,^3^ hearing loss,^4^ diabetes,^5^ and Parkinson’s disease.^6^

Next-generation neurostimulation devices aim for wireless communication with the nervous system.^7^ Among a wide variety of energy transfer options, such as ultrasound, radio frequency, and temporal interference, light allows wireless transfer of energy with high spatial and temporal resolution.^8,9^ Hence, photovoltaic biointerfaces that convert light energy to neurostimulation (i.e., photostimulation) emerged as an exciting approach for the control of neural activity.^10^ They have been used to stimulate a wide range of nerve tissues, such as motor cortex,^11^ sciatic nerve,^12^ heart,^13−15^ and retina.^16^ Also, at the clinical level they stimulate the retinal tissue and enable enhanced visual acuity for the patients with age-macular degeneration (AMD).^17^ In implants, silicon photodiodes are used and they convert the near-infrared (NIR) radiation to photovoltage to bias the electrodes.^18^ They operate in the near-infrared (NIR) window to directly communicate with the implant without any crosstalk with the surviving photoreceptors; however, the low absorption coefficient of silicon in the NIR window (e.g., 383 cm^–1^ at 880 nm) demands thick photoactive region (30 μm) making rigid implants.^19^

Colloidal nanocrystals (NCs) with inorganic crystal structures like silicon and solution processability like polymers, hold high promise for flexible bioelectronics. They offer unique features such as tunable electronic energy levels via quantum confinement effect, surface engineering via ligand exchange, and high-level of compositional variations.^20^ For neuromodulation, NCs such as CdSe, CdS, InP, and AlSb have been used for photostimulation of neurons that operate within the visible range.^21−24^ Alternatively, HgTe NCs show favorable photoresponse up to near-infrared spectral range but they are coupled with neurons via a Faradaic pathway^25^ that may lead to unwanted pH change of cellular environment.^26^ Instead, PbS NCs have demonstrated their ability to induce safe capacitive coupling with neurons.^27^ However, the destabilization of the quantum dots could potentially result in the diffusion of lead species into the body,^28^ which raises concerns about their translation to clinical devices.^29^

**Figure 1:**
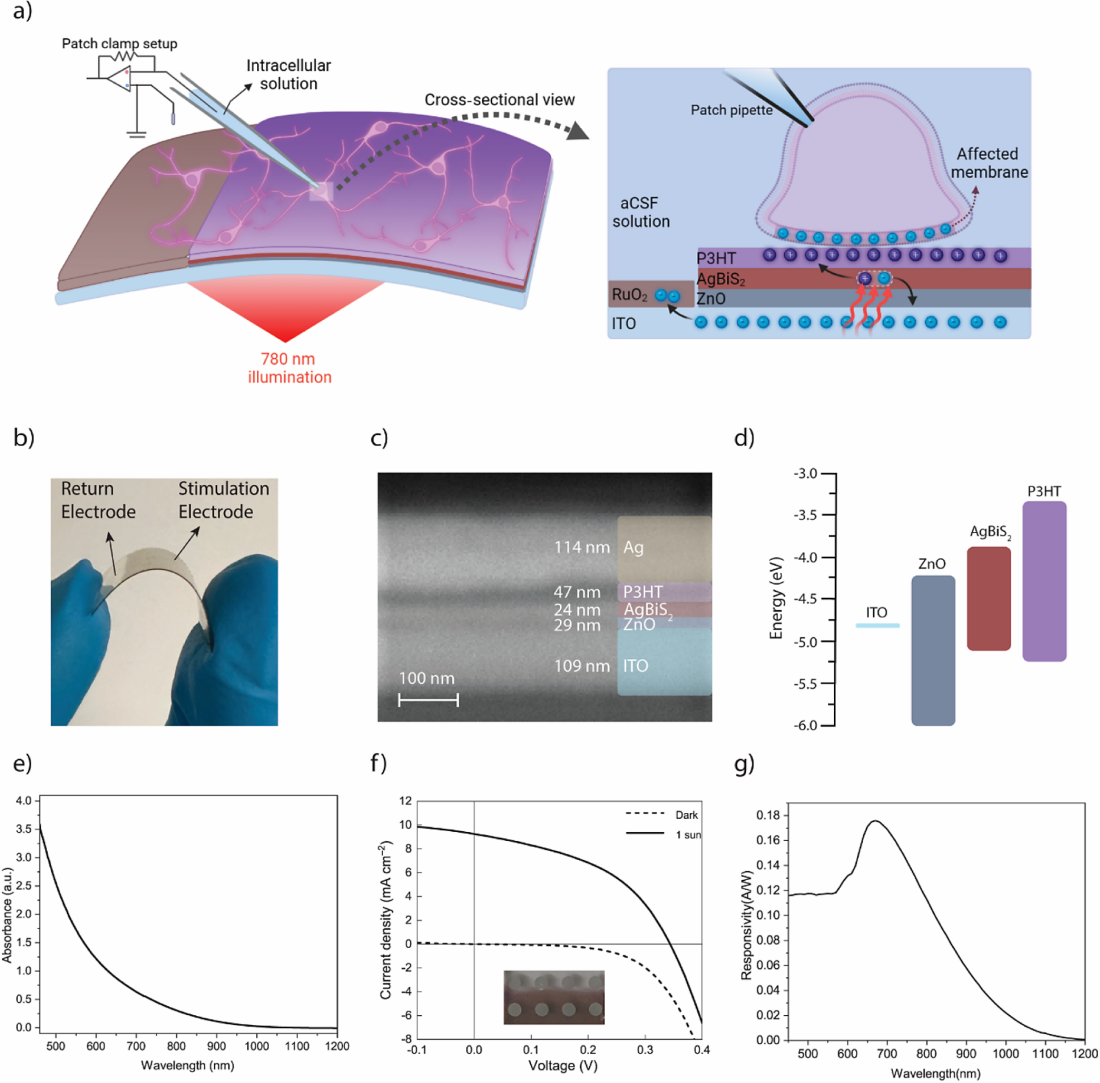
(a) Schematic of biointerface with cells cultured on top (not to scale) (left). Patch clamp electrophysiology is employed to assess the electrical activity of the cell membrane under whole cell configuration. The cross-sectional view of the coupling of biointerface with the adjacent cell (right). The stimulation electrode is composed of ITO/ZnO/AgBiS_2_/P3HT layers, and the return electrode is made of electrochemically deposited RuO_2_ layer on ITO. (b) Photograph of the flexible bioelectronic device on PET/ITO substrate. (c) Cross-sectional FE-SEM image of the biointerface. The MoOx/Ag layer is added for electrical characterization of the photodiode. (d) Energy band diagram of the photodiode. (e) Absorbance of AgBiS_2_ NCs. (f) J–V of the photodiode coated with MoOx/Ag as the contact electrode under dark and 1-sun illumination condition. (g) Responsivity of the photodiode for different wavelengths.

In this study, we demonstrate AgBiS_2_ NCs, which have no toxic heavy metal content, such as cadmium or lead, for efficient and capacitive photovoltaic neurostimulation in NIR (Figure 1a). We selected AgBiS_2_ NCs because of their relatively narrow band gap (1.1 eV) and high absorption coefficient (about 10^5^ cm^–1^) in NIR, which is stronger than other NCs including PbS, CdTe, and perovskite, and approximately 2-orders of magnitude higher than silicon.^30^ Thanks to their exceptional photoabsorption strength, we integrated a nanoscale photoactive layer into a pin diode structure that transduced high ionic photocurrents in biological medium at low-intensity levels in NIR. The devices operate without any wire connection or external battery and maintain stability in physiological conditions without any toxicity on cells. The device triggers capacitive and efficacious photostimulation of hippocampal neurons significantly below the ocular safety limits. This study reports the first toxic-heavy-metal-free nanocrystal-based optoelectronic biointerfaces operating in NIR paving the way toward retinal prosthetics.

## Results

### Bioelectronic Device and Characteristics

The photovoltaic bioelectronic device consists of two parts: stimulation and return electrodes (Figure 1a). We synthesized AgBiS_2_ NCs, and the stimulation electrode was fabricated by using a thin AgBiS_2_ NC layer (24 nm) (Figure 1b,c). NCs have oleic acid as the native ligand, but these long chains hinder efficient device production due to poor conduction. Because of that, oleic acid ligands were exchanged with tetramethylammonium iodide (TMAI).^31^ This passivation, involving stronger Bi–I and Bi–S bonds, enhanced stability, electronic properties, and performance, which led to a ∼ 10-fold increase of photocurrent (Figure S1). The energy band alignment indicates that NIR-absorbing AgBiS_2_ NCs are promising candidates to form proper heterojunction between ZnO and P3HT layers (Figure 1c,d).^31−33^ We integrated this photoactive layer between a 29 nm layer of zinc oxide (ZnO) and 47 nm poly(3-hexylthiophene) (P3HT) to promote effective exciton dissociation and photovoltage generation between the stimulation and return electrodes (Figure 1d).

The return electrode is formed via electrochemical deposition of ruthenium oxide (RuO_2_) on indium tin oxide (ITO), which can generate capacitive photocurrent.^34^ Hence, the bioelectronic device made up of ITO/ZnO/AgBiS_2_/P3HT (stimulation) and ITO/RuO_2_ (return) electrodes completes Kirchhoff’s current loop with the electrolyte. Advantageously, the entire device is flexible because of the hundreds of nanometers thick total device thickness (Figure 1b). To investigate the performance metrics of the photodiode, we added contact electrode MoOx (3 nm)/Ag (120 nm), and the IV characteristics show that the photovoltaic device works properly with an open-circuit voltage of 0.34 V and short-circuit current of 9.24 mA·cm^–2^ under 1-sun illumination (Figure 1f). The EQE of the device is 19.81% at 780 nm and has a responsivity of 0.12 A/W (Figure 1g).

### Biointerface Operation and Photoelectrochemical Characterization

The operation of the biointerface begins with the absorption of NIR light by the AgBiS_2_ NC layer. The absorption of light results in the generation of electron and hole pairs, and then, they disassociate because of the band alignment and transferred to the electron transfer (ETL) and hole transfer layers (HTL). The electrons migrate toward the lower energy states in ZnO and then move to the conductive ITO layer. Eventually, the electrons that are transferred into the ITO move to the RuO_2_ layer, which has a high electrochemical surface area/geometrical surface area (ESA/GSA) ratio that provides high interfacial capacitance.^34^ On the contrary, the holes move to the lower hole energy states toward P3HT.

**Figure 2:**
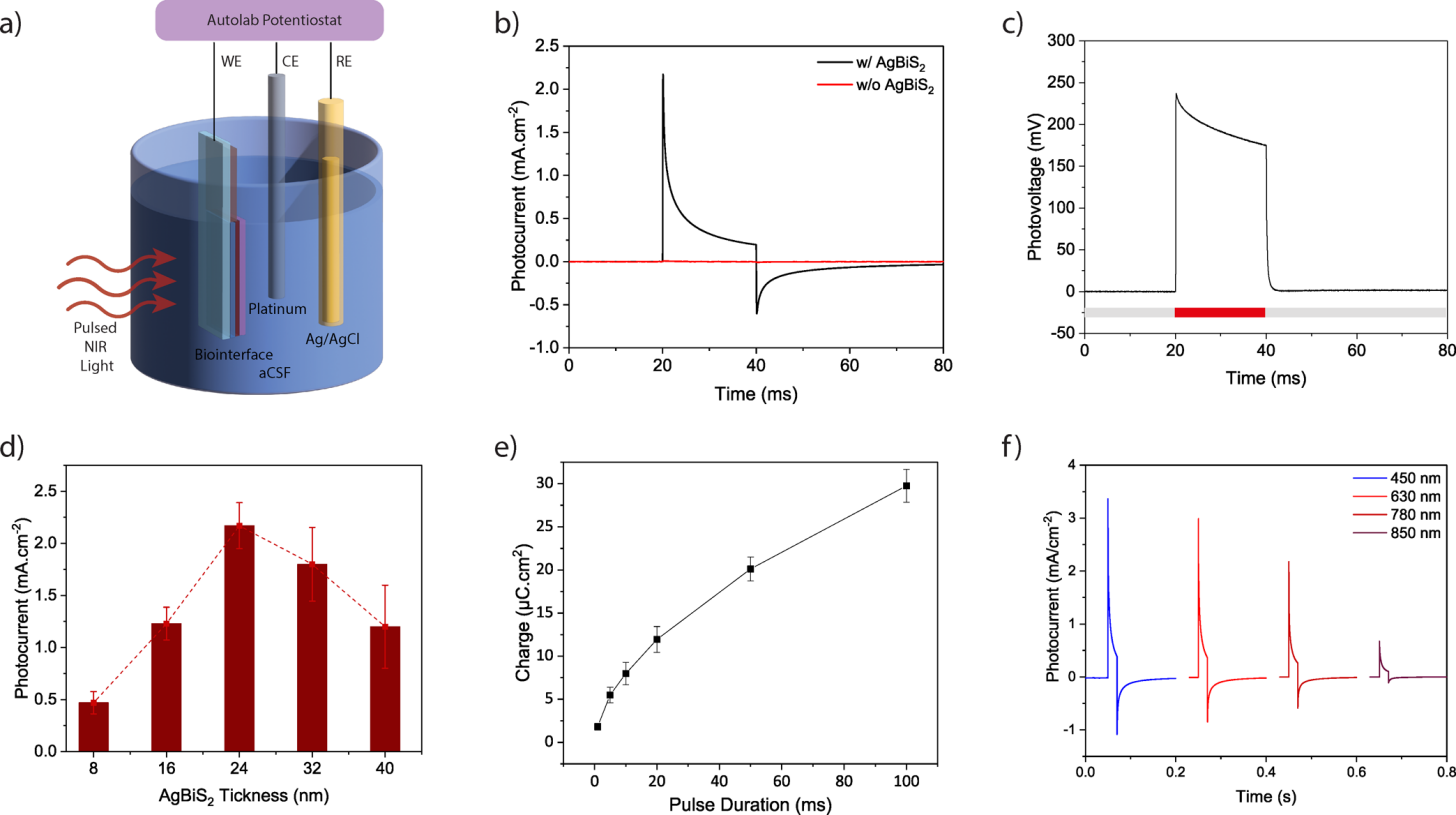
(a) Photoelectrochemistry setup used for photovoltage and photocurrent characterization of the biointerface. (b) Photocurrent response of the biointerface with ITO/ZnO/AgBiS_2_/P3HT structure (Black line) and without AgBiS_2_ (Red line) as control (Light illumination: 780 nm NIR, 20 ms, 0.5 mW·mm^–2^). (c) Photovoltage under 780 nm NIR light illumination (20 ms, 0.5 mW·mm^–2^) (mean ± SEM, *n* = 10). (d) Photocurrent peak levels for different AgBiS_2_ layer thickness under NIR light illumination (mean ± SEM, *n* = 5). (e) Charge and photocurrent with different illumination intensities using NIR light. (f) Photocurrent under different illumination wavelengths.

Three-electrode setup with an artificial cerebrospinal medium (aCSF) is used for photoelectrochemical characterization (Figure 2a). The reference electrode (RE) and counter electrode (CE) are directly dipped into an aCSF medium. The return electrode of the biointerface is connected to the working electrode (WE), and only 1 cm^2^ of the biointerface is dipped into the aCSF solution through measurements. Under 780 nm pulsed illumination, the biointerface produces a rapid capacitive peak, followed by a gradual decrease in the photocurrent profile (Figure 2b). The biointerface can generate 2.17 ± 0.2 mA·cm^–2^ (mean ± SEM, *n* = 10) and the rise time of the photocurrent is ∼51 μs, indicating a strong capacitive behavior. Due to reversible Faradaic reactions from the return electrode, photocurrent characteristics show a slow decrease after the fast-capacitive peak. This slow decrease significantly contributes to the total (ionic) charges generated in the electrolyte (i.e., the aqueous biological medium of aCSF) when the light is turned on. The biointerface can produce a photovoltage peak of 237 ± 6 mV (mean ± SEM, *n* = 10), and it can induce 10.97 ± 0.7 μC·cm^–2^ charge (mean ± SEM, *n* = 10) at 0.5 mW·mm^–2^ (Figure 2b,c).

For optimal photocurrent generation, we investigated the thickness of the AgBiS_2_ NC layer (Figure 2d). When the thickness of AgBiS_2_ is below 16 nm, we observed a significant decrease in the photocurrent, which is attributed to relatively low light absorption. When the thickness was higher than 32 nm, we observed drop of photocurrent. Hence, we used 24 nm-thick AgBiS_2_ NCs for the fabrication of the photovoltaic bioelectronic devices. Other than AgBiS_2_, each layer in the device is critical for optimal performance (Figure S2). For example, removing either ETL or HTL led to an almost 12-fold decrease in photocurrent (Figure S3) and addition of RuO_2_ increases the charge ∼ 10 times compared to the biointerface in comparison with only ITO (Figure S4). Cyclic voltammetry (CV) and electrochemical impedance spectroscopy (EIS) are utilized in aCSF to show the successful RuO_2_ coating in physiological settings (Figure S5). The symmetric and quasirectangular shapes of the cyclic voltammograms indicate the capacitive nature of the films with reversible redox reactions. Therefore, the ITO/ZnO/AgBiS_2_/P3HT architecture with RuO_2_ return electrode led to the high photoelectrical performance in terms of capacitive response and charge injection.

Charge generation level is another important criterion for effective stimulation of neurons, and as the light intensity increases, the ionic charge injection increases (Figure 2e). The device can generate 5.9 μC·cm^–2^ even at low light levels of 0.1 mW·mm^–2^ at NIR, which is sufficiently high for neurostimulation in vivo.^35,36^ Moreover, we investigated photoresponse for different wavelengths (Figures 2f and S6), and the biointerface generates a larger photocurrent of 450 nm because of the absorption of the P3HT layer in blue and the higher oscillator strength of AgBiS_2_ NCs toward shorter wavelengths. The absorbance spectrum of the P3HT also shows that there is almost no absorption in the NIR region (Figure S7), which proves that our device is operating under the NIR with the contribution only by the AgBiS_2_ NC layer (Figure S8).

**Figure 3:**
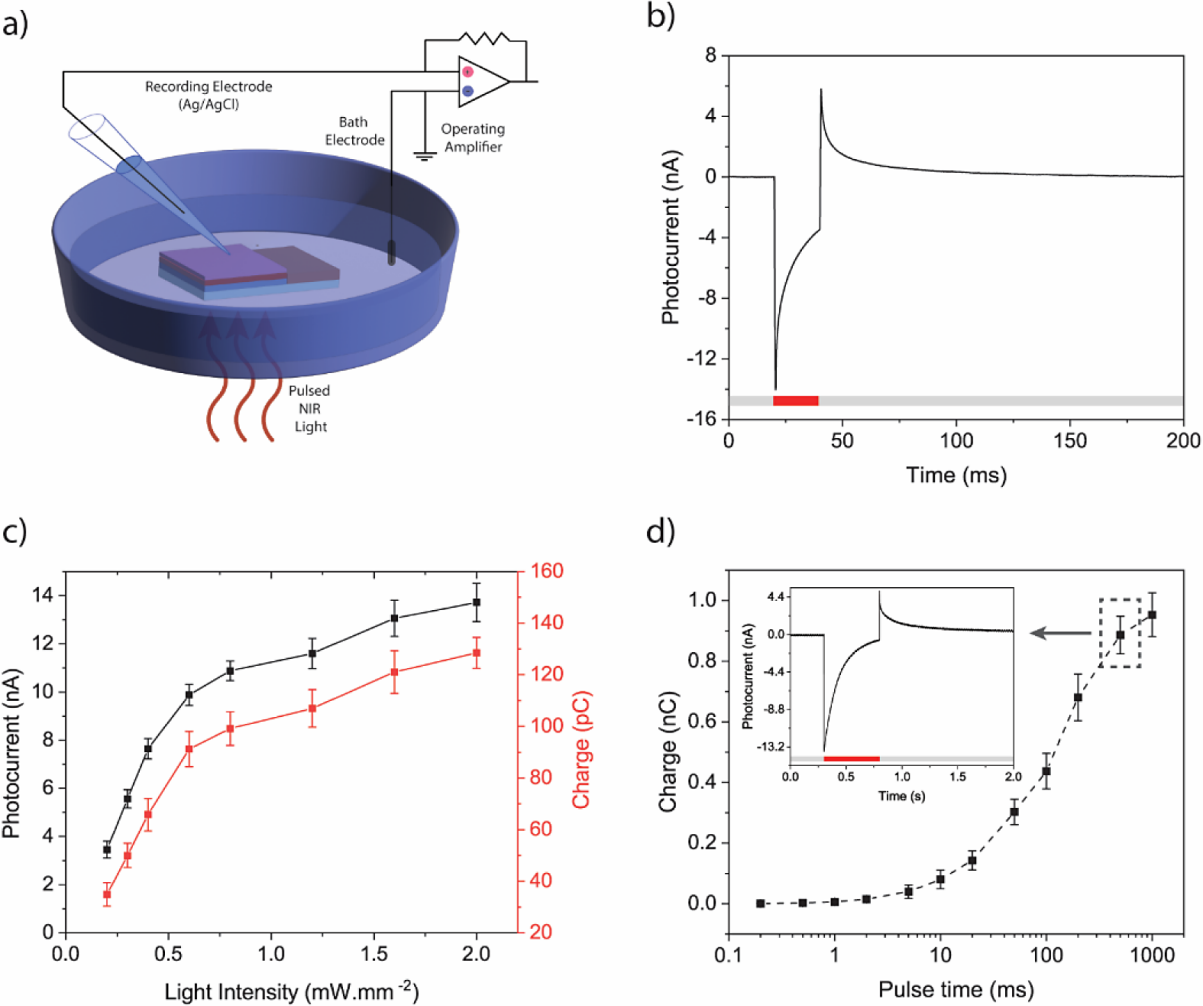
(a) Photocurrent measurement via patch-pipette tip positioned close to the surface of the biointerface. (b) Photocurrent of the biointerface under illumination. (c) Photocurrent and charge of the biointerface depending on light intensity using 780 nm light illumination (mean ± SEM, *n* = 10). (d) Charge depending on pulse duration of the illumination (mean ± SEM, *n* = 10).

We further investigated the photocurrent and charge generation of the biointerface in a stand-alone and wireless mode, similar to the working condition in cellular environments. A glass capillary as the counter electrode and a distant Ag/AgCl bath electrode as the reference were used to measure the photocurrent performance (Figure 3a). We measured the photocurrent with the illumination power of 2 mW·mm^–2^ and obtained 13.7 ± 0.8 nA (mean ± SEM, *n* = 10), with a high-level of charge injection confirming the electrochemical measurements (Figure 3b). To better understand the charge injection amount for different pulse configurations, we applied illuminations ranging from 1 to 1000 ms at 0.1 Hz (Figure 3d). We observed that as the pulse duration increases the charge significantly rises as expected. Moreover, we observed less than 3% contribution by the faradaic contribution (e.g., 500 ms) indicating capacitive dominant charge transfer mechanism.^11^

### Stability and Biocompatibility

**Figure 4:**
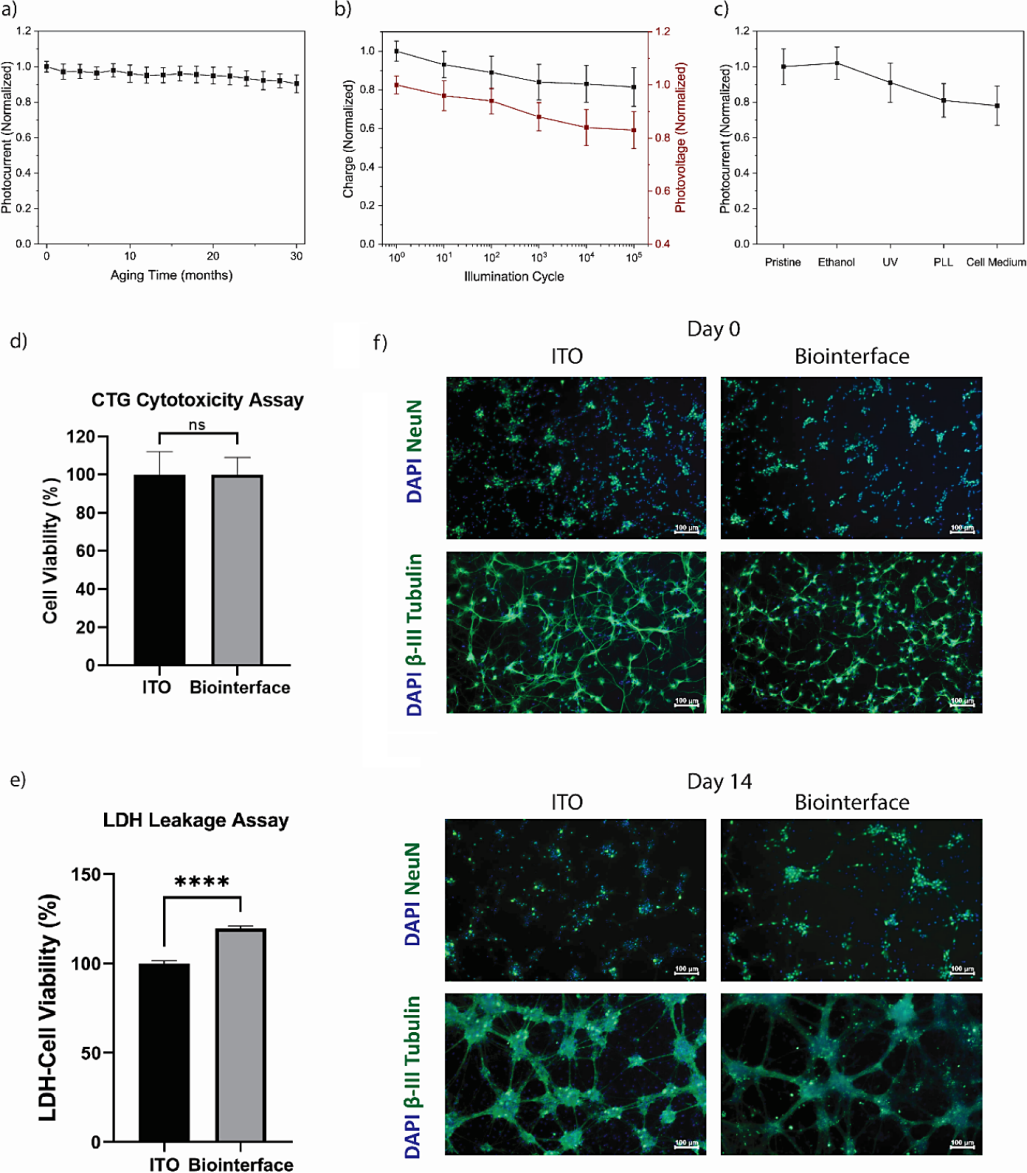
(a) Photocurrent under passive accelerated aging test. Biointerfaces were placed in physiological saline solution at 87 °C in dry oven under dark conditions for 680 h to age for 30 months. Chronopotentiometry measurements were repeated in each 45 h (mean ± SEM, *n* = 4). (b) Photocyclic stability of the biointerface (mean ± SEM, *n* = 4) under the stimulus frequency of 100 Hz, pulse-width of 5 ms, optical power density of 2.5 mW·mm^–2^. (c) Sterilization stability test, under ethanol dipping, UV sterilization, PLL coating, keeping in cell medium (3-day incubation). (d) CTG cytotoxicity assay for quantifying cell viability of primary hippocampal neurons cultured on the ITO control and the AgBiS_2_ biointerface (mean ± SEM, *n* = 4). (e) LDH leakage assay for measuring cell viability through cell membrane integrity of neurons on the substrates (mean ± SD for *n* = 5). An unpaired, two-tailed *t* test was used for statistical analysis, and *p < 0.05 was evaluated as statistically significant. (f) Immunofluorescence images at day 0 and day 14 of neurons cultured on the ITO and biointerface. Cells were costained with DAPI (blue) nuclear marker and Anti-NeuN (green) neuronal nuclear marker or costained with DAPI (blue) nuclear marker and Antibeta III Tubulin (green) neuronal structure marker (scale bar: 100 μm).

We investigated the photostability and biocompatibility of the devices without any encapsulation. For the long-term stability of the biointerface, we applied a passive accelerated aging test. This involves placing the samples into a glass Petri dish filled with H_2_O_2_-supplemented aCSF and exposing them to 87 °C oven under dark conditions for 680 h to simulate the aging for 30 months.^37^ The photocurrent was measured using chronoamperometry every 45 h simulating 30 months of aging (Figure 4a). As a result, we observed a halftime of 12.5 years. We also tested the photostability of the device under long illumination cycles. Here, the charge and photovoltage remained around 80% compared to the initial values after 10^5^ repetitive photoexcitation (Figure 4b).^11^ This behavior highlights the capacitive nature of the device, and high charge injection from the return electrode is due to reversible reactions. Before checking the biocompatibility of the devices, we explored the effect of sterilization steps, which consisted of a 5 min ethanol dip, UV treatment, PLL coating, and overnight cell medium incubation (Figure 4c). The photocurrent results indicate that the performance of the device is mainly preserved after sterilization tests.

Since in our previous studies we demonstrated that the use of P3HT on the biointerface had no adverse effect on cell survival,^38,39^ at first we tested the effect of AgBiS_2_ NCs on primary hippocampal cells by using devices without the P3HT layer. Morphological features of neurons during the 14-day culture period were maintained on ITO/ZnO/AgBiS_2_ devices and showed no significant alteration in morphology with respect to the control group (Figure S9). Then, CTG cell viability and LDH leakage analysis were performed to examine the cytotoxicity of neurons growing on ITO/ZnO/AgBiS_2_/P3HT with RuO_2_. CTG test provides the cell viability depending on the amount of ATP in the cell populations, while LDH leakage test measures the release of lactate dehydrogenase (LDH) from cells in the culture environment. CTG measurement showed high viability of primary hippocampal neurons cultured on the ITO control and biointerface (Figure 4d). We observed that the biointerfaces had no negative effect on the viability of primary hippocampal neurons via the LDH leakage test, which is consistent with CTG results. The amount of LDH leakage from cells cultured with the device was lower compared to the reference ITO control group, indicating a high level of cell viability (Figure 4e).

The short-term and long-term effects of the substrates on the cells (at day 0 and day 14, respectively) were monitored in terms of number of neurons, their characteristics, and morphology. NeuN and beta-III-Tubulin staining was performed to examine the neuronal specific properties and functional changes. The cells remained healthy and viable during 14 days of the culture period and conserved their specific characteristics with enlarged neuronal network both on ITO control and biointerface (Figure 4f). DAPI and NeuN positive cells were analyzed to show the number of neuron and total cells per unit area, and they remained similar throughout the 2-week culture period (Figure S10). Neurite length analysis was carried out using antibeta-III-Tubulin staining, during which neurites were traced from the perimeter of the soma to their end points. Cells and the growth of neurites exhibited similar distribution on both ITO and the biointerface. In the first week of culture, the neurite extensions of the neurons on the ITO appear to significantly longer compared to the biointerface; however in the following days, it was observed that the connections between the axon extensions and cell populations were enhanced in the biointerface compared to ITO (Figure S11).

### Single-Cell Electrophysiology Recordings

**Figure 5:**
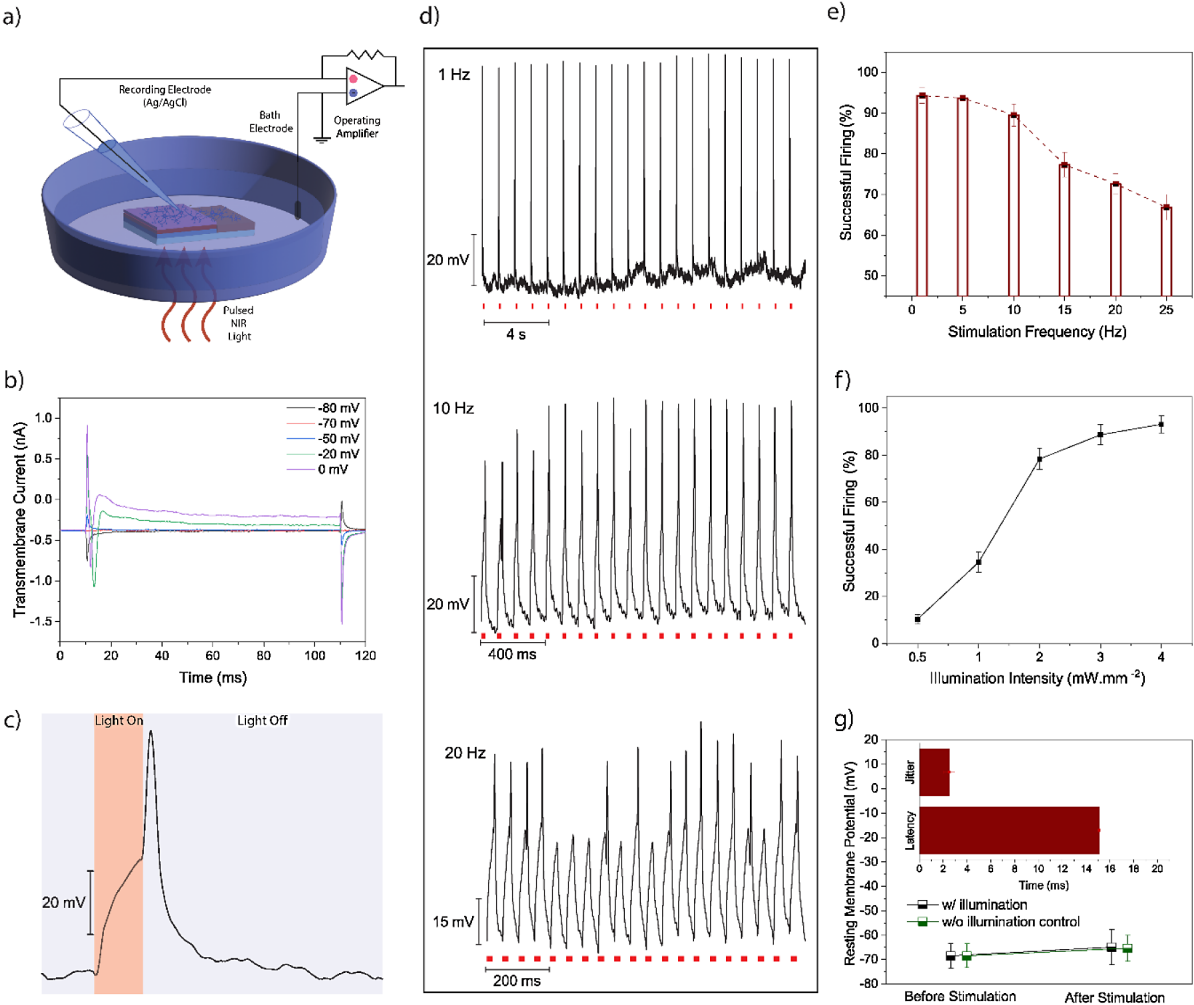
(a) Single-cell patch-clamp electrophysiology recording of hippocampal neurons on the biointerface. (b) Transmembrane current recording in whole-cell configuration by applying different holding potentials. (c) A typical action potential of neurons on the biointerface under 780 nm light illumination. (d) Single cell electrophysiology recordings for different frequencies 1, 10, and 20 Hz (from top to down, respectively), with same pulse width of 20 ms. (e) Successful action potential firing versus stimulation frequency at 2.5 mW·mm^–2^ (mean ± SEM, *n* = 4 neurons). (f) Action potential firing versus illumination intensity under NIR illumination at 5 Hz (mean ± SEM, *n* = 4 neurons). (g) Resting membrane potential before and after stimulation. Inset: Spike latency and jitter of neuronal membrane induced by the biointerface (mean ± SEM, *n* = 4 neurons).

We cultured primary hippocampal neurons on the biointerface to show light-induced neural stimulation. The primary neurons are well-accepted in vitro models to analyze action potential activities.^40^ We utilized patch clamp electrophysiology experiments to record the electrical activity of neurons on the biointerface under NIR illumination. First, the intracellular potential of the neurons is measured from the soma, where the reference Ag/AgCl bath electrode is placed inside the same extracellular solution distant from the biointerface (Figure 5a). The cells remained in the patch for an average of 5 ± 4 min (mean ± SEM, *n* = 10) without a significant change in the resting membrane potential. We recorded the transmembrane current under whole cell patch in voltage clamp mode (Figure 5b) and observed fast sodium current at −30 mV holding potential indicating that 40 mV of depolarization is required to excite the cell.

We record action potentials when the depolarization passes the threshold level (Figure 5c). The photogenerated holes are confined close to the surface of the biointerface and this positive charge induces hyperpolarization of the attached membrane while the recorded free membrane has depolarization.^41^ Stimulation frequency is important for the successful firing of a neuron, where a firing probability of 94.3 ± 1.9% (mean ± SEM, *n* = 10) is observed for 1 Hz illumination at an intensity level of 2.5 mW·mm^–2^. For 25 Hz illumination, it decreases to a 66.8 ± 3.1% (mean ± SEM, *n* = 10) (Figure 5d,e). While we change the intensity, the successful firing probability also varies. For example, the biointerface induces a successful firing of 78.4 ± 4.4% (mean ± SEM, *n* = 10) at 1.5 mW·mm^–2^ (Figure 5f), and when we decrease the intensity to 1 mW·mm^–2^, the device is still able to trigger firing of neurons, which correspond to over an order of magnitude lower than the ocular safety limit at 780 nm (i.e., 18.75 mW·mm^–2^).

To understand the temporal precision of neural stimulation, we analyzed the mean latency (time between stimulation onset and action potential peak) and jitter (standard deviation of latencies for all neurons; Figure 5g inset). We found that the mean latency is 15.0 ± 3.6 ms (mean ± SEM) and jitter is 2.5 ± 0.4 ms (mean ± SEM, *n* = 10). Considering recent methods like photothermal and photoacoustic stimulation, our latency value is much smaller and comparable with the ranges reported in optogenetics.^42−44^ In addition, we applied 1000 pulses with a 5 Hz illumination frequency, and there was no significant change in resting membrane potential compared to control devices (Figure 5g). Since the maximum temperature increase with 2.5 mW·mm^–2^ illumination is just 0.12 °C,^45^ thermal increase does not have any significant contribution to the stimulation. We also observed no depolarization of neurons by the ITO control substrates under illumination (Figure S12). Thus, the device can operate within the safe limits under pulsed NIR light exposure (Figure S13).^46^

## Discussion

NIR photostimulation of neurons is facilitated by AgBiS_2_ nanocrystals contaning the green heavy metal of bismuth,^47^ whereas toxic lead, mercury, and cadmium heavy metal-based NCs were used in previous reports.^20^ Since AgBiS_2_ NCs have absorption coefficient higher than other inorganic photovoltaic materials, such as silicon, GaAs, and InP, in the NIR window ranging from 750 to 900 nm,^32^ such a strong absorption enabled an ultrathin bioelectronic interface and the use of small quantities of nanomaterials (∼0.5 μg). Furthermore, the reduced thickness serves the additional purpose of minimizing the occupied volume of implants, which can potentially facilitate seamless integration.

The efficient photocurrent generation is critical to control the stimulation contrast of the retina while operating below the ocular safety limit. In terms of quantum dots, this device, which has around 4-fold higher photocurrent than the previous PbS-based optoelectronic biointerfaces,^27^ has the best light to electricity conversion reported device performance in NIR so far^20^ (Table S1). Like quantum dots, organic semiconductors, such as indigo dyes, polymers, and silicon nanostructures, are exciting materials for flexible photovoltaic neurostimulation devices, but they generally operate in the visible spectrum up to deep red.^48−50^ Only one pioneering recent study using an bulk heterojunction photovoltaic device extended the spectral window toward NIR.^51^

Diseases that lead to degeneration of the retina such as retinitis pigmentosa and age-related macular degeneration (AMD) are the primary cause of the permanent loss of vision. NIR responsivity of the bioelectronic interfaces is critical to communicate with the retinal prosthetics implanted into patients.^18^ Patients with the atrophic form of AMD have surviving photoreceptors that allow them to have peripheral vision. Since photoreceptors have around 5-orders-of-magnitude less sensitivity at the wavelength used in this study in comparison with the peak at 555 nm,^52^ NIR illumination can directly facilitate photostimulation of neurons without any interference with the remaining photoreceptors, potentially enabling perception of artificial vision.^17^ The flexibility of such devices can open the possibility of large-area implementation into the retina, resulting in an extended field of view, which is advantageous for mobility and performing visually driven search tasks of patients.^53^

## Conclusion

In conclusion, we demonstrated the first toxic heavy-metal-free nanocrystal-based optoelectronic biointerface operating within the NIR spectral window. The extraordinary absorption strength of AgBiS_2_ NCs allowed efficient photocurrent generation via a nanoscale NC layer (i.e., 24 nm). At the same time, the devices made of AgBiS_2_ showed good biocompatibility and stability in the cellular environment. Patch-clamp electrophysiology of primary hippocampal neurons enabled us to observe the modulation of membrane potential. These devices can induce successful photostimulation of primary hippocampal neurons at tens of hertz below the ocular safety limits. Hence, AgBiS_2_ nanocrystals show high potential for future retinal prosthetics.

## Methods

### Synthesis of AgBiS_2_ Nanocrystals

AgBiS_2_ nanocrystals (NCs) were synthesized by following previously reported hot-injection method using standard Schlenk techniques with small modifications.^31^ All chemicals were purchased from Sigma-Aldrich. In brief, 6.4 mmol of silver(I) acetate and 8 mmol of bismuth(III) acetate were degassed with 136 mmol of oleic acid and 35 mL of 1-octadecene (ODE) with raising the temperature. After reaching 100 °C, reaction vessel was maintained in vacuum environment for 1 h to generate silver and bismuth oleates. 8 mmol bis(trimethylsilyl)sulfide (TMS) solution diluted in 5 mL of ODE was swiftly injected into the reaction vessel after switching the reaction atmosphere to Ar. The color of solution was immediately changed to dark brown, indicating the formation of AgBiS_2_ NCs. To conduct the purification process of AgBiS_2_ NCs, the reaction flask was cooled in water bath, and the crude solution was precipitated with acetone using a centrifuge at 4500 rpm for 5 min. Sediments were collected and reprecipitated with toluene and acetone and this purification cycle was repeated two times. Resulting AgBiS_2_ NCs were finally dispersed in toluene with a concentration of 20 mg/mL to fabricate the bioelectronic interfaces.

### Characterization of AgBiS_2_ Nanocrystals

The UV–vis absorption measurement of the solution was carried out with a Cary 5000 spectroscopy. The XRD measurements were conducted by a Rigaku SmartLab powder diffractometer using Cu Kα radiation with Bragg–Brentano geometry. TEM measurements were performed at the Scientific and Technological Centers of the University of Barcelona (CCiT-UB). TEM micrograph was measured by a JEOL 2100 microscope operating at an accelerating voltage of 200 kV.

### Fabrication of Biointerface

Photoelectrodes were fabricated on glass/ITO (Osilla, S111) and PET/ITO (Sigma-Aldrich). The substrate cleaning procedure consists of sonication in NaOH solution for 5 min, tension-active agent mixed with deionized water solution (HELLMANEX II, 3%) for 15 min, deionized water for 15 min, pure acetone for 5 min, and isopropyl alcohol for 5 min, all at 55 °C. The cleaned substrates were treated with UV-ozone for 20 min to eliminate any other possible residues on the ITO surface. For ZnO layer coating, ZnO precursor solution was prepared by mixing 219.3 mg zinc acetate dehydrate (Zn (CH_3_CO_2_)_2_·_2_H_2_O) from Sigma-Aldrich in 2 mL of 2-methoxyethanol (C_3_H_8_O_2_) and 90 mg of ethanolamine (HOCH_2_CH_2_NH_2_) and sonicated for 25 min at 50 °C. Then, the ZnO solution was filtered by a 0.45 μm PVDF filter. The ZnO layer was spin coated onto the ITO substrates at 2000 rpm for 60 s and annealed at 280 °C for 20 min for glass and 200 °C for 1 h for PET. Afterward, three layers of AgBiS2 NCs were deposited from 20 mg·mL^–1^ toluene solution via the layer-by-layer method. For each AgBiS2 NC layer, 50 μL of AgBiS2 NC solution was spin coated onto ITO/ZnO substrates during spinning (2000 rpm). Then, TMAI/methanol (1% v/v) solution was applied to the NC film for 45 s, followed by two rinse–spin steps with methanol and once with toluene. The films were transferred into the glovebox for 10 min annealing at 115 °C. The P3HT (95.7% regioregular) (>99% pure, Ossilla) was utilized without any further purification. Photoactive solution was prepared as a 20 mg·mL^–1^ solution of P3HT in o-dichlorobenzene and stirred overnight at 70 °C. The P3HT layer was fabricated onto the ZnO layer by spin coating at 1500 rpm for 60 s and annealed at 150 °C for 10 min. For characterizations and neuro-related experiments, biointerfaces that are fabricated on glass substrates were used.

### Fabrication of Solar Cells

After the biointerface fabrication, 3 nm of MoO_3_ and 120 nm of Ag were evaporated sequentially through a shadow mask to produce solar cells with a diameter of 2 mm (area of 3.1 mm^2^) using a Kurt J. Lesker Nano36 system. The devices were exposed to air in the dark for at least 1 day before measuring.

### Characterization of Solar Cells

Cross-sectional scanning electron microscopy (cross-SEM) image of the AgBiS_2_ solar cell was obtained using a JSM-7100 F JEOL (Toko, Japan) instrument from the Centres Cientfics i Tecnolgics de la Universitat de Barcelona (CCiTUB). The solar cell performance was characterized by current–voltage (J–V) measurement using a Keithley 2400 source meter and solar simulator (AM 1.5 G, Newport Oriel Solar 3A Class AAA) after calibration of light intensity using a Hamamatsu S1336 silicon photodiode. The external quantum efficiency (EQE) spectrum was collected with Newport Cornerstone 260 monochromator, a Thorlabs MC2000 chopper, a Stanford Research SR570 transimpedance amplifier, and a Stanford Research SR830 lock-in amplifier after calibration using Newport 818-UV photodetector as a reference.

### Chronoamperometry and Chronopotentiometry Measurements

For the electrochemical experiments, we used an Autolab Potentiostat Galvanostat PGSTAT (Metrhom, The Netherlands). The three-electrode configuration utilizes Ag/AgCl as the reference electrode, platinum wire as the counter electrode, and the connection to the biointerface as the working electrode. All measurements were carried out in a modified aCSF medium as the electrolyte solution at room temperature. The device was excited with blue and red LEDs. The optical power was controlled with an optical power meter (Newport 843-R). The data were analyzed using the NOVA software.

### Photocurrent Measurements

The measurements were carried out using an Olympus T2 upright microscope and extracellular patch clamp (EPC) 800 patch clamp amplifiers (HEKA Elektronik GmbH, Pfalz, Germany). The modified aCSF aqueous medium was prepared by mixing 10 mM of 4-(2-hydroxyethyl)-1- piperazineethanesulfonic acid (HEPES), 10 mM of glucose, 2 mM CaCl_2_, 140 mM of NaCl, 1 mM of MgCl_2_, 3 mM of KC, and distilled water. The pH was calibrated to 7.4 using 1 M NaOH. As the illumination source, Thorlabs’ blue (M450LP1) was used. LED system was driven by DC2200 High-Power 1-Channel LED Driver with Pulse Modulation (Thorlabs Inc., NJ, USA). Photocurrent was measured without electrical grounding of the ITO layer, and the ground was connected to the electrolyte solution to simulate the biological environment.

### Preparation of Substrates for Primary Neuron Culture

Each substrate of the control ITO and the biointerfaces were rinsed with 70% ethanol before being left to dry out completely. Dried substrates were placed on the 6-well plates. Then, UV sterilization was carried out for 30 min under the cell culture hood. After sterilization, substrates were coated with poly-l-lysine (PLL, Sigma-Aldrich, MO, USA) to increase cell attachment to the substrates for 4 h in a 37 °C, 5% CO_2_ cell culture incubator. After incubation, the substrates were rinsed with Nanopure water and left to dry under a culture hood before seeding the primary neurons.

### Primary Neuron Isolation

All experimental procedures have been approved by the Institutional Animal Care and Use Committees of Koç University (Approval No: 2021.HADYEK.022) according to Directive 2010/63/EU of the European Parliament and of the Council on the Protection of Animals Used for Scientific Purposes. For primary neuron isolation, the hippocampi tissue was dissected from the brains of embryos of Wistar Albino rats on embryonic day E16–E18. The tissues were placed in a falcon tube containing ice-cold Hank’s Balanced Salt Solution (HBSS) immediately after the dissection. After being rinsed with ice-cold HBSS, they were incubated in 0.25% Trypsin-EDTA solution with 2% DNase-I supplement in a falcon tube for 20 min at 37 °C to initiate enzymatic digestion. The tube was rotated up and down 2–3 times to get better digestion. To terminate the enzymatic reaction, Dulbecco’s Modified Eagle Medium/Nutrient Mixture F-12 (DMEM/F12) enriched with 10% fetal bovine serum (FBS) and 1% penicillin/streptomycin was added to the tube. Then the tissue suspension was centrifuged at 300*g* for 3 min. The suspension including DMEM/F12 was carefully discarded and replaced with Neurobasal Plus medium (NBM) enriched with B27, l-glutamine, B-mercaptoethanol, glutamate, and 1% penicillin/streptomycin. The NBM added cell pellet triturated continuously with a plastic Pasteur pipet until no large tissue pellet were visible. After triturating, the homogeneous cell solution flowed through the 70-μm cell strainer into the new falcon tube. The cells from the falcon tube were seeded onto each poly-l-Lysin-coated substrate of control ITO and biointerfaces. The substrates were then settled in 37 °C, 5% CO_2_ cell culture incubator for 3 days. On the third day, half of the entire medium of the cells was changed with the NBM-enriched Cytosine Arabinoside to prevent glial cell overgrowth. After 24 h incubation, all of the media inside the cell was replaced with complete Neurobasal Medium (NBM) supplemented with B27, l-glutamine, and 1% penicillin/streptomycin for substrates to be ready for future experiments. For 14 days of incubation, half of the medium of the cells was changed every 3–4 days.

### Cell-Titer Glo Toxicity Assay

CellTiter-Glo Luminescent Cell Viability Assay (CTG, Promega, Mannheim, Germany), which shows the presence of adenosine triphosphate (ATP) in metabolically active cells, was used to determine the cell viability of primary neurons on the substrates of ITO and the biointerface. Cells (500,000 cell/well) were seeded on the samples and incubated for 3 days. On the third day of isolation (DIV3), half of the entire medium of the cells was changed with the NBM-enriched Cytosine Arabinoside to prevent glial cell overgrowth as described above. After 24 h of incubation, medium was replaced with complete neurobasal medium (NBM) and the next day at day 0 (DIV5) CTG viability test performed. Before starting the experiment, the substrates were removed from the incubator and left at room temperature for 30 min, then CellTiter-Glo Reagent was added to each substrate. The plate was put on the orbital shaker for 5 min and incubated for 10 min in the RT. After incubation, each medium of substrates was placed on a 96-well opaque plate, and the luminescence signals were measured with Synergy H1Microplate Reader (Bio-Tek Instruments). The relative cell viability of the sample was determined by comparing it with that of the control ITO.

### Lactate-Dehydrogenase Release Assay

CytoSelectTM LDH Cytotoxicity Assay Kit (CBA-241, Cell Biolabs) was performed to examine the cell membrane integrity of primary neuronal cells growing on the samples. For the LDH leakage test, cells (500,000 cells/well) were seeded as described above, and at day 0 (DIV5) the LDH leakage was evaluated. Reference control ITO substrates were used, along with positive and negative controls. In order to induce LDH leakage as a positive control, cells on ITO were incubated with culture medium containing 1% Triton-X-100 for 10 min at 37 °C in a 5% CO_2_ cell culture incubator. For the negative control, incubation was carried out with an equal amount of water. Incubation with a medium including 1% water was also applied to the experimental groups. After 10 min, 90 μL of culture medium was transferred from each sample to transparent 96-well cell culture plates. Subsequently, 10 μL of LDH cytotoxicity assay reagent was added to each well, and the plates were incubated at 37 °C in a 5% CO_2_ incubator for 30 min to allow the reaction to occur. The amount of LDH leaked into the culture medium was measured at a 450 nm optical density using the Synergy H1Microplate Reader (BioTek) device. The relative cell viability of the biointerface from the LDH leakage rate was determined by comparing it with the control ITO.

### Immunofluorescence Staining and Imaging

Primary hippocampal neurons were seeded as explained above on the ITO control and the AgBiS_2_ biointerfaces with 500,000 cells/well. The neurons at day 0 or day 14 were fixed by ice cold 4% paraformaldehyde in PBS for 20 min and washed three times with PBS-T (Phosphate Buffered Saline, 0.1% Tween-20). For permeabilization, the cells were incubated with 0.1% TritonX-100 in PBS for 8 min followed by blocking with Superblock (Thermo Fisher Scientific, MA, USA) for 10 min at RT. To check neuron morphology and characterization, cells were labeled overnight with anti-NeuN antibody (ab177487, Abcam, Cambridge, UK) and an anti-*b*-III Tubulin antibody (ab18207, Abcam, Cambridge, UK) prepared in a blocking solution. After incubation, samples were washed with PBS-T, and they were incubated with goat antirabbit IgG H&L Alexa Fluor 488 (Cell Signaling Technology, MA, USA) for 90 min at 37 °C. All samples were washed three times with PBS-T, and mounted with DAPI supplemented mounting medium (50001, Ibidi GmbH, Germany). All immunofluorescence images were taken by inverted fluorescence microscope (Axio Observer Z1, ZEISS, Oberkochen, Germany). For day 14 experiments, the same procedure was performed on the 14th day of culture.

### Electrophysiology Experiment

Experiments were performed with an EPC 800 patch clamp amplifier (HEKA Elektronik GmbH, Pfalz, Germany). Biointerfaces and control devices were cleaned with 70 vol % ethanol solution and incubated for 3 days in DI water. The pulled patch pipettes of 4–6 MΩ were used to carry out the whole-cell patch clamp experiment. Extracellular medium (modified aCSF) was prepared as described in the photocurrent measurement section. The internal cellular medium was prepared by mixing 140 mM KCl, 2 mM MgCl_2_, 10 mM HEPES, 10 mM ethylene glycol-bis(β-aminoethyl ether)-N,N,N′,N′-tetraacetic acid (EGTA), 2 mM Mg-ATP in water and the pH was calibrated to 7.2–7.3 using 1 M KOH. The electrophysiology experiments were performed around DIV 5–7. The osmolarity of internal solution was >270 mOsm, and the osmolarity of external solution was <290 mOsm. Patch pipettes were filled with the intracellular solution to achieve whole-cell patch, where all the experiments were performed at resting membrane potential. A digital camera integrated Olympus T2 upright microscope was used to patch and monitor the cells. The blue LED (M450LP1, Thorlabs Inc., NJ, USA) was used as the light source. LED system was driven by DC2200-High-Power 1-Channel LED Driver with Pulse Modulation (Thorlabs Inc., NJ, USA).

### Optical Safety Considerations

The maximum permissible radiant power (MP) that can be chronically delivered to the retina was calculated according to the ocular safety standards.^46^ The photochemical limit does not apply in the NIR region, and the equation for photothermal and photoacoustic limit is MP = 6.93 × 10^–5^ CT CE P–1. CT = 100.002 (λ-700) = 1.445 for λ = 780 nm. CE was taken as 29.3 W·mm^–2^ considering a retinal spot size larger than 1.7 mm in diameter in accordance with a previous study.^40^ The equation for the single-pulse limit for the pulse-widths between 0.05 and 70 ms is given as MP_single_ = 6.93 × 10^–4^ CT CE t–0.25. These equations give average irradiance limits and the peak irradiance limits can be calculated from MP_peak_ = MP_avg_/(*t* × *f*), where *t* is pulse duration and *f* is pulse frequency.

## Supporting Information Available

Photocurrent of the biointerface without applying the TMAI treatment; photocurrent measurements for different P3HT and ZnO layer thickness; photocurrent in the absence of ZnO electron transfer layer; photocurrent in the presence and absence of RuO_2_; cyclic voltammogram and electrochemical impedance measurement of RuO_2_; photocurrent under different illumination wavelengths; absorbance of ITO/ZnO/P3HT; absorbance of ITO/ZnO/AgBiS_2_/P3HT; performance comparison table with previous reports; bright field and immunofluorescence images neurons cultured on the ITO and ITO/ZnO/AgBiS_2_ devices; immunofluorescence images and quantification of number of cells; immunofluorescence images and quantification of neutrite length of cells; single cell electrophysiology recordings of cells on control devices; permissible light exposure based on ocular safety standards; the X-ray diffraction (XRD) spectrum and transmission electron microscopy (TEM) image of AgBiS_2_ NCs (PDF)
