# Supplementary material for: Capacitive and Efficient Near-Infrared Stimulation of Neurons via an Ultrathin AgBiS2 Nanocrystal Layer
Source: ACS Appl Mater Interfaces. 2024 May 29;16(23):29610–20. doi: 10.1021/acsami.4c01964 (PMC11661670; doi:10.1021/acsami.4c01964)
Supplement: Supplementary file 1 — am4c01964_si_001.pdf [file am4c01964_si_001.pdf]

Supporting Information

Capacitive and Efficient Near-infrared Stimulation of Neurons via Ultra-thin $AgBiS_2$ Nanocrystal Layer

Ridvan Balamur[1], Jae Taek Oh[2], Onuralp Karatum[1], Yongjie Wang[2], Asim Onal[3], Humeyra Nur Kaleli[4], Cigdem Pehlivan[4], Afsun Şahin[4], Murat Hasanreisoglu[4], Gerasimos Konstantatos[2,5], Sedat Nizamoglu[1,*]

[1]*Department of Electrical and Electronics Engineering, Koç University, Istanbul, 34450, Turkey*

[2]*ICFO-Institut de Ciencies Fotoniques, The Barcelona Institute of Science and Technology, Castelldefels, 08860, Barcelona, Spain*

[3]*Department of Biomedical Science and Engineering, Koç University, Istanbul, 34450, Turkey*

[4] *Research Center for Translational Medicine, Koç University, Istanbul, 34450, Turkey*

[5] *ICREA - Institució Catalana de Recerca i Estudiats Avançats, Lluis Companys 23, 08010 Barcelona, Spain.*

*snizamoglu@ku.edu.tr

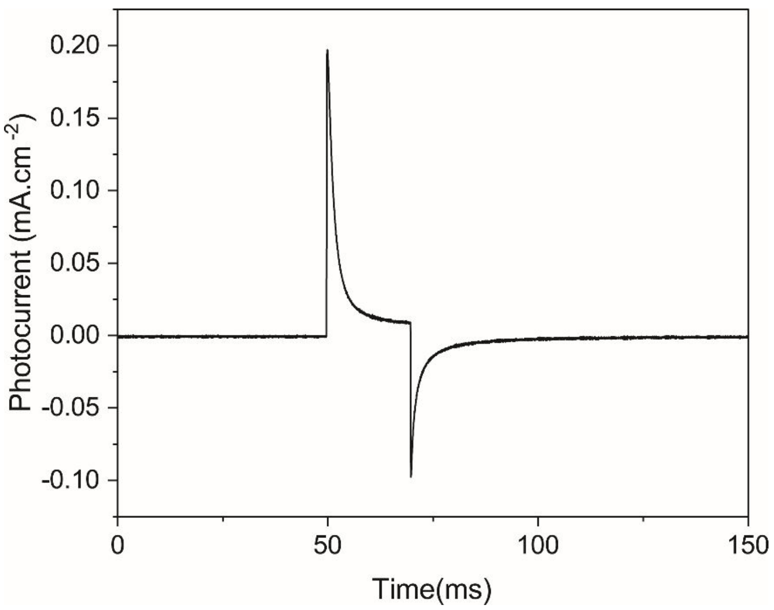

Figure S1 Photocurrent of the biointerface without applying the TMAI treatment. The biointerface has the following structure: ITO/ZnO/$AgBiS_2$/P3HT with RuO_2 in the return electrode. Illumination condition: 20 ms, 780 nm, 0.5 mW.mm^{-2}.

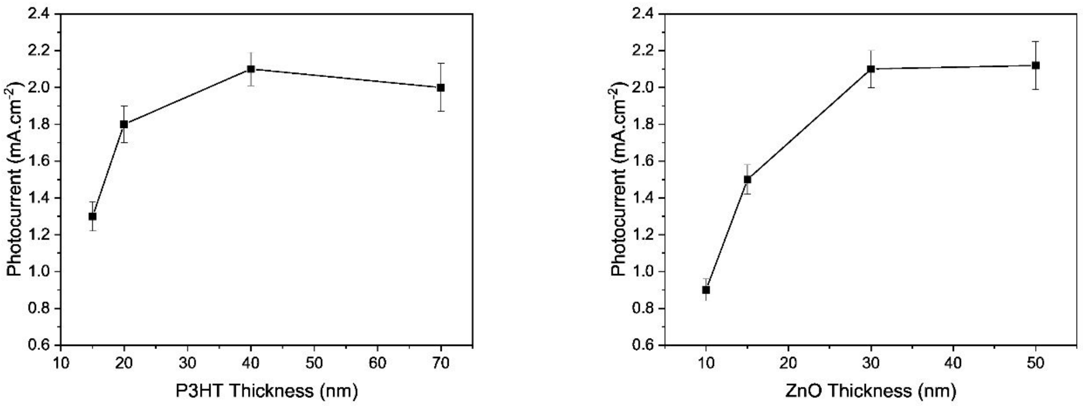

Figure S2 Photocurrent measurements for different P3HT (a) and ZnO (b) layer thickness under NIR light illumination (mean ± SEM, n=5).

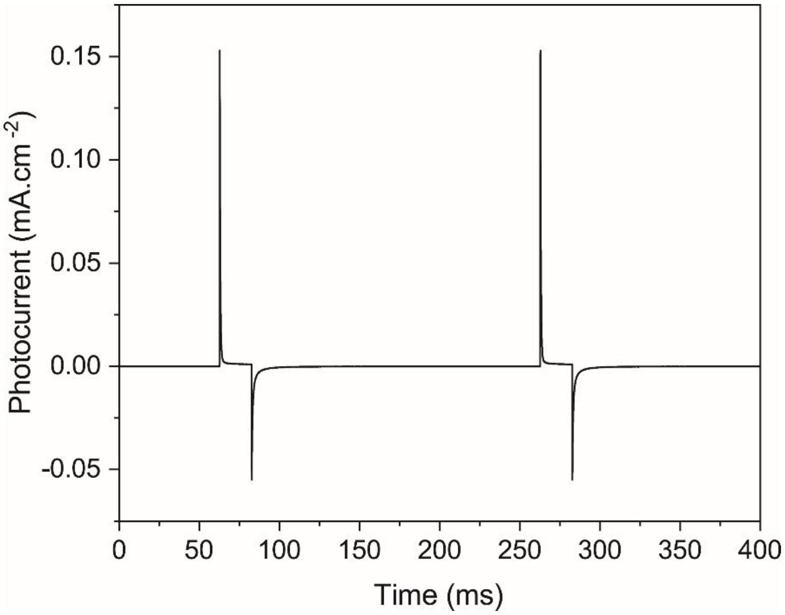

Figure S3 Photocurrent in the absence of ZnO electron transfer layer. The biointerface has the following structure: ITO/$AgBiS_2$/P3HT with ITO in the return electrode. Illumination condition: 20 ms, 780 nm, 0.5 mW.mm^{-2}.

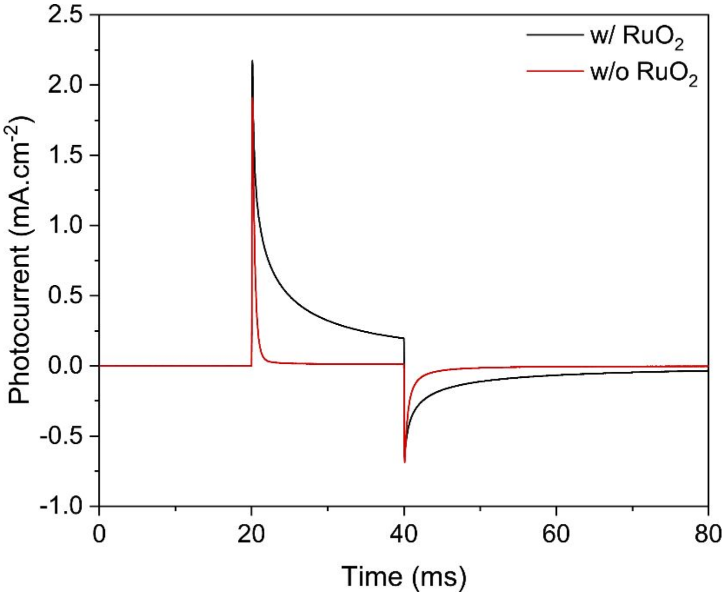

Figure S4. Photocurrent in the presence and absence of RuO_2 on the return electrode. Illumination condition: 20 ms, 780 nm, 0.5 mW.mm^{-2}.

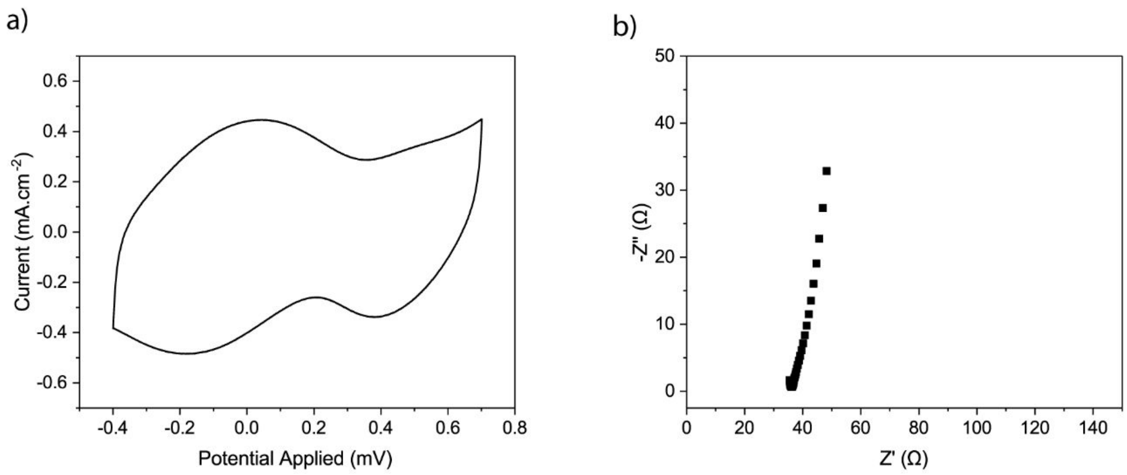

Figure S5 a Cyclic voltammograms of RuO_2 coating from −0.4 to 0.7 V range. Scan rate is 50 mV s^{-1}. **b** Electrochemical impedance measurement of RuO_2 coatings within the frequency range of 1–10 000 Hz.

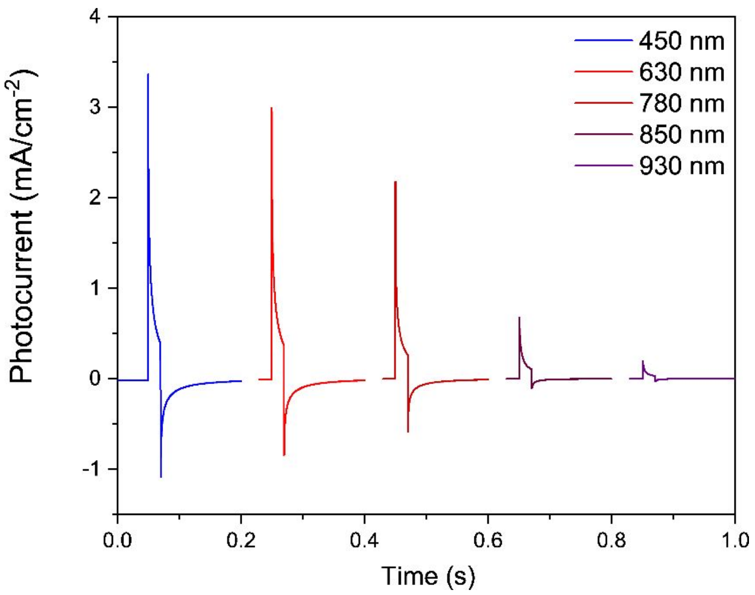

Figure S6 Photocurrent under different illumination wavelengths.

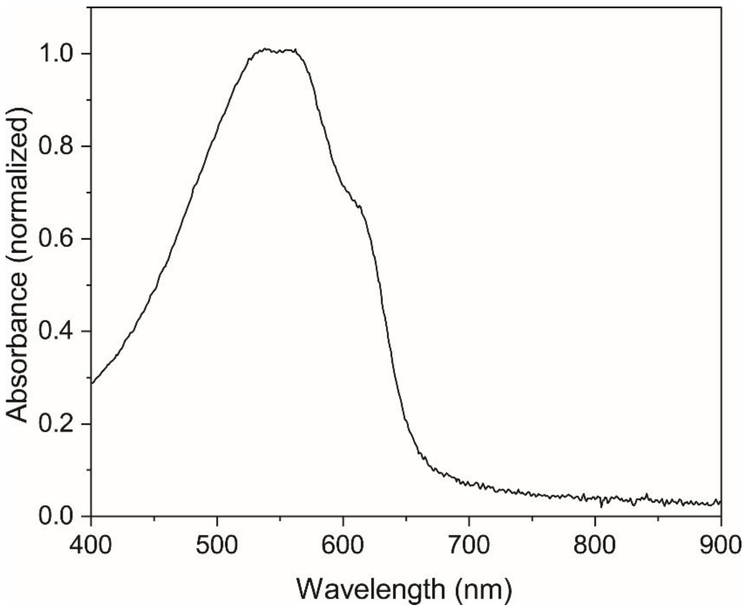

Figure S7. Absorbance of ITO/ZnO/P3HT.

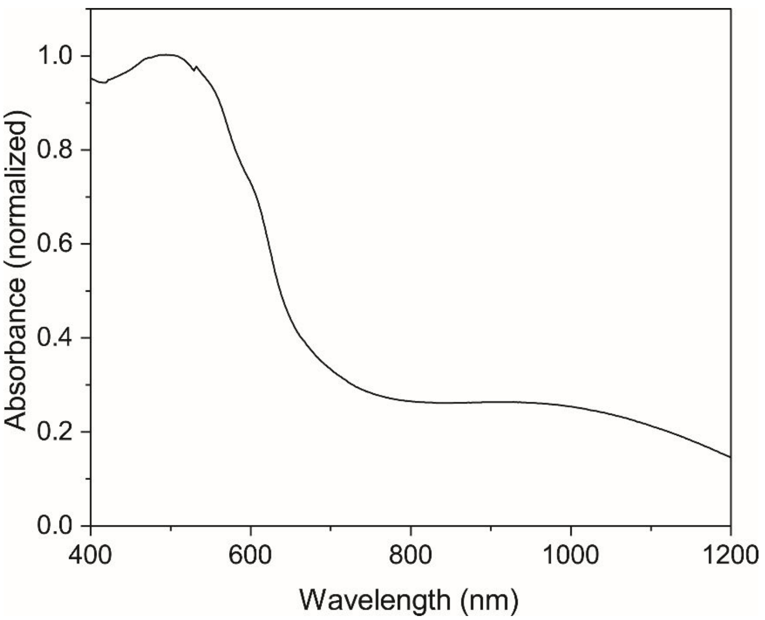

Figure S8. Absorbance of ITO/ZnO/$AgBiS_2$/P3HT showing that absorbance in NIR range is due to the contribution of $AgBiS_2$ NC layer.

Table S1. Active layer thicknesses, photocurrent, and operational wavelengths from previous reports compared to this study.

References	Active layer Thickness	Photocurrent	Operational wavelength
**This study**	**24 nm**	**2.3 $mA.cm^{-2}$**	**780 nm**
[1]	30 nm	800 $\mu A.cm^{-2}$	630 nm
[2]	60 nm	2 $mA.cm^{-2}$	638 nm
[3]	25 nm	0.5 $mA.cm^{-2}$	780 nm
[4]	30 µm	9.2 $mA.cm^{-2}$	880 nm

We monitored the short-term effects of the ITO and ITO/ZnO/$AgBiS_2$ substrates on the cells at day 0 and tracked long-term alterations in neuron characteristics and morphology up to the 14th day of culture (DIV19). Bright field images and β-III-Tubulin staining was utilized to assess neuronal-specific properties. Throughout the 14-day culture period, the cells remained robust and viable, retaining their specific characteristics, and exhibiting an expanded neuronal network, both on the ITO control and the device (**Figure S9**). Images were taken by confocal laser scanning microscope (DMI8 SP8, Leica, Wetzlar, Germany).

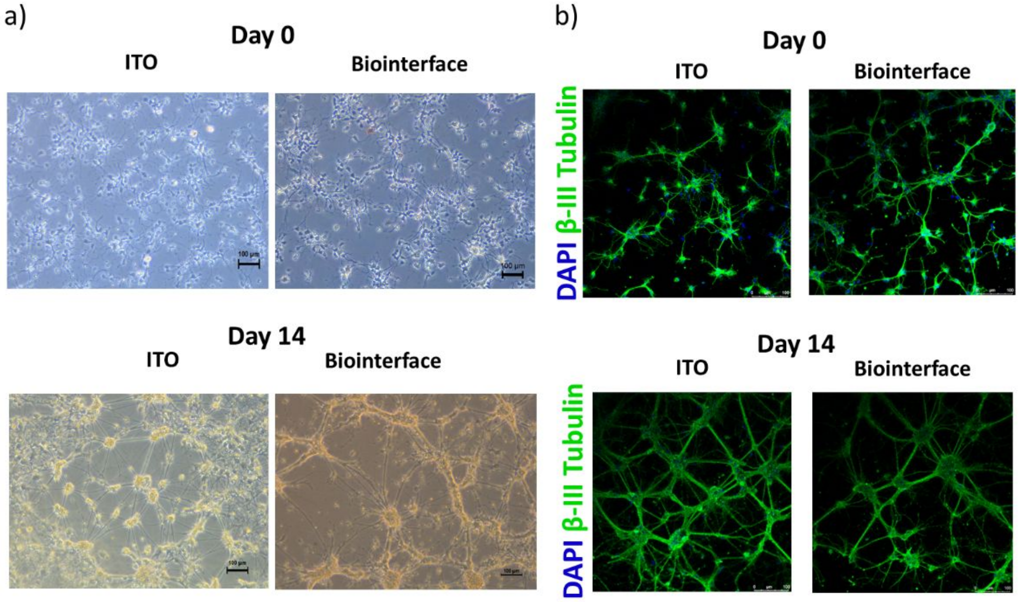

Figure S9 a Bright field images of neurons on ITO control and biointerface during 2 weeks of the culture period (scale bar: 100 µm) and **b** Immunofluorescence images at Day 0 and Day 14 of neurons cultured on the ITO and ITO/ZnO/$AgBiS_2$ devices. Cells were co-stained with DAPI (blue) nuclear marker and anti-β-III-Tubulin (green) neuronal structure marker (scale bar: 100 µm).

The cell number/mm^2 counting: DAPI and NeuN positive cells were analyzed to show the number of neuron and total cells per unit area. 10X objective images were used for the counting, the image size is 0.713 mm by 1.13 mm with an area of 0.805 mm^2. The neuron cells were counted as the number of NeuN positive cells per area (neuron counts/mm^2) and the total cells were counted as the number of DAPI positive cells per area (neuron counts/mm^2) in ImageJ [5]. There is not any significance found in number of neuron and total cells between control and biointerface at Day 0 and Day 14. Ordinary one-way ANOVA was used to determine the statistical significance of the differences among multiple groups, and $*p < 0.05$ was evaluated as statistically significant.

The mean number of neurons and total cells at Day 0 on ITO was counted as 753.1 ± 182.4 (mean ± SD, n=4); 1024 ± 240.5 (mean ± SD, n=4) while on biointerface was 742.2 ± 34.01 (mean ± SD, n=4); 804.3± 71.44 (mean ± SD, n=4). The mean number of neurons and total cells at Day 14 on ITO was counted as 579.5 ± 83.97 (mean ± SD, n=4); 749.7 ± 107.7 (mean ± SD, n=4) while on biointerface was 664.0 ± 107.7 (mean ± SD, n=4); 1036 ± 175.0 (mean ± SD, n=4) (Figure S10).

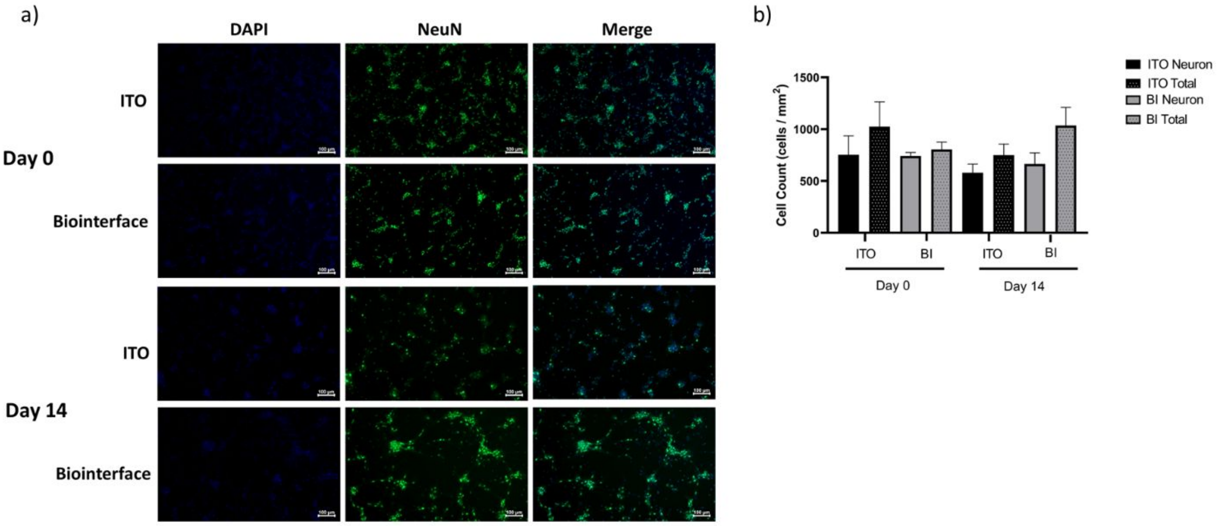

Figure S10 a Immunofluorescence images at Day 0 and Day 14 of neurons cultured on the ITO and biointerface. Cells were co-stained with DAPI (blue) nuclear marker and Anti-NeuN (green) neuronal nuclear marker. **b** Quantification of the number of NeuN positive cells and total number of cells per mm^2 for each culture period shown in immunofluorescence images (mean ± SD, n =4)

Neurite Length Measurement: Neurite length analysis was performed by anti-beta-III Tubulin staining using NeuronJ plugin program in ImageJ. The length of neurite was measured by manually tracing a neurite from the boundary of the soma to the end of neurite. The average of 50 cells from each group were used in calculation. Ordinary one-way ANOVA was used to determine the statistical significance of the differences among multiple groups, and *$p < 0.05$ was evaluated as statistically significant [6, 7].

In the first week of culture, the neurite extensions of the neurons on the ITO appear to significantly longer as 274.2 ± 83.36 μm (mean ± SD, n=50) compared to the biointerface 197.1 ± 69.10 μm (mean ± SD, n=50). In the following days, it was observed that the axon extensions and cell populations were enhanced in the biointerface 199.7 ± 60.67 μm (mean ± SD, n=50) compared to ITO 163.0± 52.73 μm (mean ± SD, n=50).

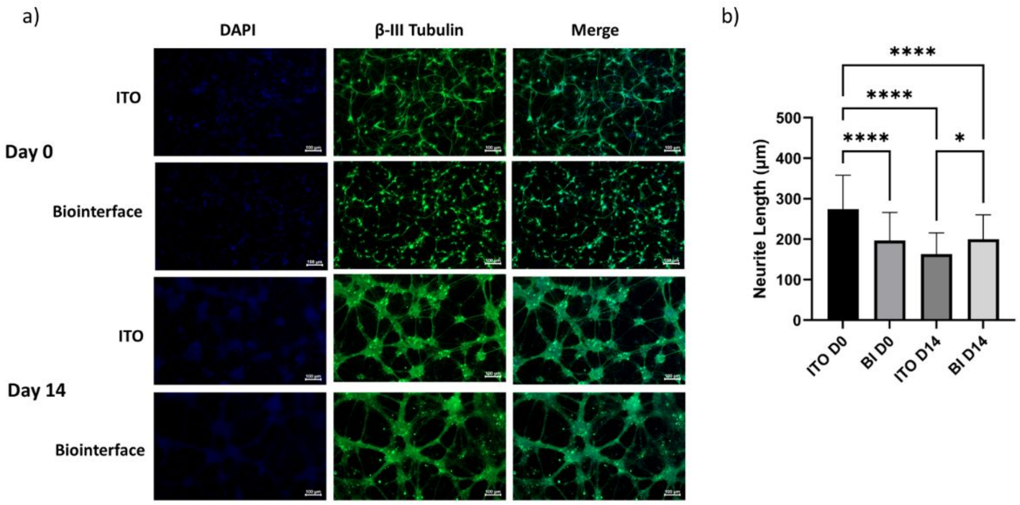

Figure S11 a Immunofluorescence images at Day 0 and Day 14 of neurons cultured on the ITO and biointerface. Cells were co-stained with DAPI (blue) nuclear marker and Anti-beta III Tubulin (green) neuronal structure marker. **b** Quantification of neurite length for each culture period were performed from immunofluorescence images (mean ± SD, n = 50 cells)

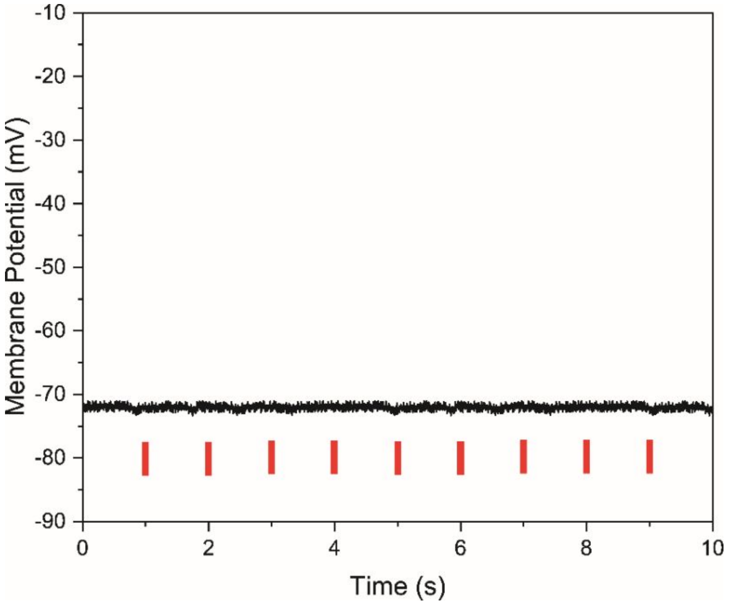

Figure S12 Single cell electrophysiology recordings measured from the cells cultured on control ITO devices. The frequency of illumination is 1 Hz.

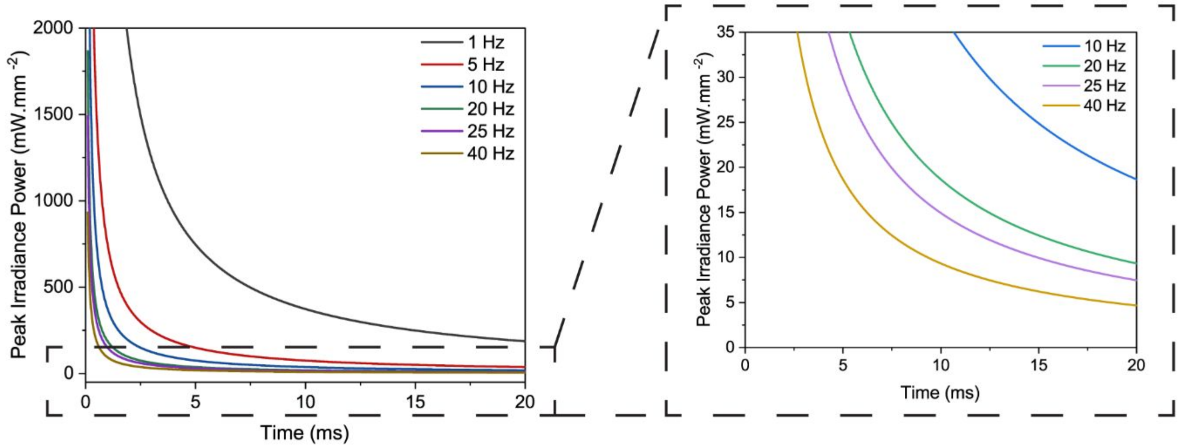

Figure S13. Calculated permissible light exposure based on ocular safety standards [8] for different pulse durations and frequencies at 780 nm.

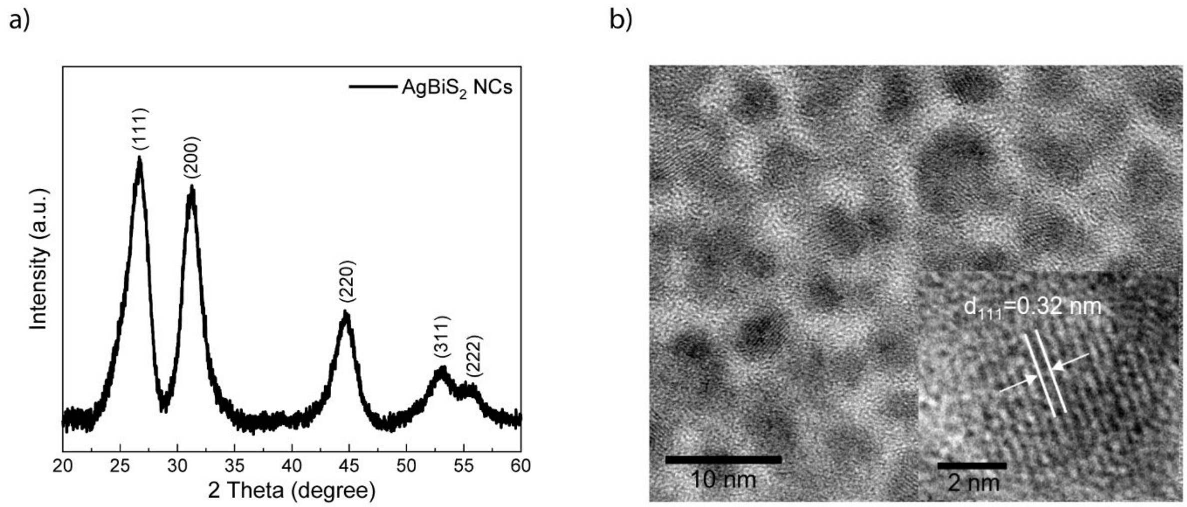

Figure S14 a The X-Ray diffraction (XRD) spectrum and **b** transmission electron microscopy (TEM) image of $AgBiS_2$ NCs.

Referances:

1. Silverå Ejneby, M., et al., *Extracellular photovoltage clamp using conducting polymer-modified organic photocapacitors.* Advanced Materials Technologies, 2020. **5**(3): p. 1900860.
2. Silverå Ejneby, M., et al., *Chronic electrical stimulation of peripheral nerves via deep-red light transduced by an implanted organic photocapacitor.* Nature Biomedical Engineering, 2022. **6**(6): p. 741-753.
3. Karatum, O., et al., *Electrical stimulation of neurons with quantum dots via near-infrared light.* Acs Nano, 2022. **16**(5): p. 8233-8243.
4. Huang, T.W., et al., *Vertical-junction photodiodes for smaller pixels in retinal prostheses.* Journal of neural engineering, 2021. **18**(3): p. 036015.
5. Cevallos, C., et al., *Bystander Effects and Profibrotic Interactions in Hepatic Stellate Cells during HIV and HCV Coinfection.* Journal of Immunology Research, 2024. **2024**.
6. Meijering, E., et al., *Design and validation of a tool for neurite tracing and analysis in fluorescence microscopy images.* Cytometry Part A: the journal of the International Society for Analytical Cytology, 2004. **58**(2): p. 167-176.
7. Pemberton, K., B. Mersman, and F. Xu, *Using ImageJ to assess neurite outgrowth in mammalian cell cultures: research data quantification exercises in undergraduate neuroscience lab.* Journal of Undergraduate Neuroscience Education, 2018. **16**(2): p. A186.
8. Delori, F.C., R.H. Webb, and D.H. Sliney, *Maximum permissible exposures for ocular safety (ANSI 2000), with emphasis on ophthalmic devices.* JOSA A, 2007. **24**(5): p. 1250-1265.
